# Impact of Methicillin Resistance on Outcomes of *Staphylococcus aureus* Bacteremia at a U.S. Center, 2023–2024

**DOI:** 10.1093/ofid/ofag354

**Published:** 2026-06-06

**Authors:** Joseph B Ladines-Lim, Aidan Nemiroff, Leigh Cressman, Michael Z David

**Affiliations:** Division of Infectious Diseases, Department of Medicine, Penn Medicine, University of Pennsylvania, Philadelphia, Pennsylvania, USA; Leonard Davis Institute of Health Economics, University of Pennsylvania, Philadelphia, Pennsylvania, USA; Center for Clinical Epidemiology and Biostatistics, Perelman School of Medicine, University of Pennsylvania, Philadelphia, Pennsylvania, USA; The College of Arts and Sciences, University of Pennsylvania, Philadelphia, Pennsylvania, USA; Center for Clinical Epidemiology and Biostatistics, Perelman School of Medicine, University of Pennsylvania, Philadelphia, Pennsylvania, USA; Division of Infectious Diseases, Department of Medicine, Penn Medicine, University of Pennsylvania, Philadelphia, Pennsylvania, USA; Center for Clinical Epidemiology and Biostatistics, Perelman School of Medicine, University of Pennsylvania, Philadelphia, Pennsylvania, USA; Department of Biostatistics and Epidemiology, Perelman School of Medicine, University of Pennsylvania, Philadelphia, Pennsylvania, USA

**Keywords:** bacteremia, clinical outcomes, MRSA, MSSA, *Staphylococcus aureus*

## Abstract

**Background:**

Methicillin-resistant *Staphylococcus aureus* (MRSA) bacteremia has historically been associated with worse outcomes than methicillin-susceptible *S. aureus* (MSSA), but MRSA epidemiology has evolved and contemporary data are mixed. We evaluated whether methicillin resistance is independently associated with *S. aureus* bacteremia (SAB) outcomes.

**Methods:**

We conducted a retrospective cohort study of adults with a first-episode SAB at two academic hospitals from January 17, 2023 to January 16, 2024. The primary outcome of in-hospital mortality/hospice discharge was compared between MRSA and MSSA. Secondary outcomes included intensive care unit (ICU) admission, 7-day readmission, metastatic complications, infective endocarditis, and recurrent bacteremia. Multivariable logistic regression was used to estimate adjusted odds ratios (aORs), with propensity score matching as sensitivity analysis.

**Results:**

Among 329 patients with SAB, 140 (42.6%) had MRSA and 189 (57.4%) had MSSA. Compared with MSSA, MRSA was associated with younger age, lower body mass index, higher injection drug use prevalence, more skin and soft tissue sources, and lower illness severity. After covariate adjustment, methicillin resistance was not associated with mortality/hospice discharge (aOR 1.03, 95% CI 0.49–2.12), ICU admission (aOR 1.09, 95% CI 0.61–1.94), 7-day readmission (aOR 1.51, 95% CI 0.51–4.67), metastatic complications (aOR 0.98, 95% CI 0.47–2.05), infective endocarditis (aOR 1.02, 95% CI 0.42–2.45), or recurrent bacteremia (aOR 2.43, 95% CI 0.92–6.76). Findings were consistent in propensity-matched analyses.

**Conclusions:**

In this contemporary academic cohort, methicillin resistance was associated with distinct patient characteristics but not worse short-term outcomes. Host factors, illness severity, and infection source may be more important prognostic determinants.

## Teaser

In a contemporary academic cohort of adults with *Staphylococcus aureus* bacteremia, methicillin resistance was associated with distinct epidemiologic characteristics but not worse short-term outcomes. Host factors, illness severity, and infection source may better inform prognosis than resistance phenotype alone.

## Graphical Abstract

**Graphical Abstract:**
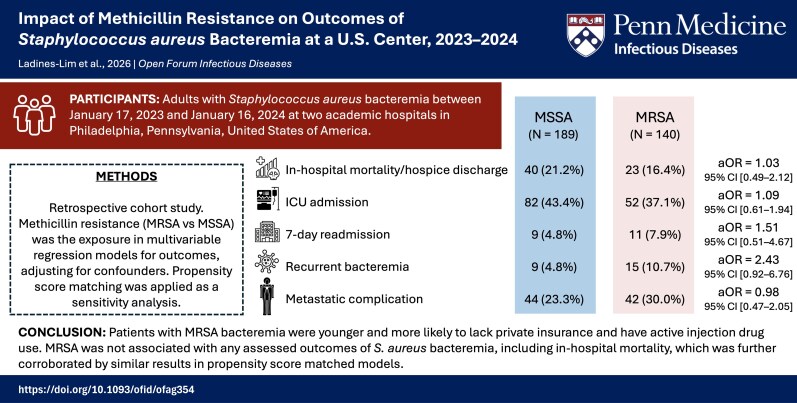

*Staphylococcus aureus* bacteremia (SAB) remains one of the most consequential bacterial infections worldwide, accounting for an estimated 300 000 deaths annually with case fatality rates ranging from 15% to 30% [1]. In addition, SAB frequently results in metastatic complications, including infective endocarditis, osteomyelitis, septic arthritis, and other deep-seated infections, contributing to prolonged hospitalization and healthcare utilization [1, 2].

Historically, bacteremia caused by methicillin-resistant *S. aureus* (MRSA) has been associated with worse outcomes than methicillin-susceptible *S. aureus* (MSSA). Prior studies have reported higher mortality, longer hospital length of stay, increased intensive care unit (ICU) admission, and greater risk of recurrence among patients with MRSA bacteremia compared with MSSA bacteremia [2–9]. As a result, methicillin resistance has long been viewed as an independent marker of disease severity and adverse prognosis in SAB.

During the past two decades, however, the epidemiology of MRSA has evolved substantially. Once predominantly a healthcare-associated pathogen, MRSA now increasingly causes infections in the community, with changing patient demographics, risk factors, and infection sources [10–18]. Concurrently, advances in the clinical management of SAB, including earlier recognition and infectious disease consultation, standardized diagnostic evaluation, prompt source control, and expanded antimicrobial options [1, 19–21], may have mitigated historical differences in outcomes between MRSA and MSSA bacteremia. In this context, contemporary studies have yielded mixed results, with several reporting no difference in mortality or other outcomes after accounting for patient characteristics and illness severity [9, 22–24].

Despite these changes, methicillin resistance continues to influence clinical decision-making and risk stratification in SAB [1]. Whether MRSA remains independently associated with adverse outcomes in contemporary practice, or whether previously observed differences primarily reflect changes in host factors and epidemiology, remains uncertain. Furthermore, few recent studies have examined whether methicillin resistance is associated with specific complications, such as infective endocarditis.

To address these questions, we conducted a retrospective cohort study of adults with first-episode SAB at two academic hospitals, evaluating the association between methicillin resistance and clinical outcomes such as mortality. We hypothesized that MRSA bacteremia would be associated with distinct epidemiologic characteristics, but not worse short-term outcomes compared with MSSA bacteremia.

## METHODS

### Study Setting and Population

We performed a retrospective cohort study of sequential adults with a first episode of SAB between January 17, 2023 and January 16, 2024 at two academic hospitals within a single healthcare system in Philadelphia, Pennsylvania: the Hospital of the University of Pennsylvania (HUP) and Penn Presbyterian Medical Center (PPMC), which have 1069 and 347 beds, respectively. Eligible subjects were adults aged ≥18 years with ≥1 blood culture positive for *S. aureus*. We only included the first episode of bacteremia during the study period.

### Exposure and Covariates

The exposure was methicillin resistance status, which we determined based on Clinical and Laboratory Standards Institute guideline-concordant testing of index blood culture isolates, categorized as MRSA or MSSA. Through manual abstraction of patient records, we obtained baseline subject characteristics, including demographics, body mass index (BMI), source of infection, length of infection (defined as number of days with cultures positive for *S. aureus*), choice of antibiotic agent(s) for definitive therapy (defined by which agent had the most number of days of therapy), statin use, anticoagulant or aspirin use, active injection drug use (IDU), presence of indwelling intravascular catheter or foreign body, whether any procedure performed, prior *S. aureus* infection (identified by any culture positive for *S. aureus* from any anatomic site), history of immunosuppression in 6 months prior to infection, and variables necessary to determine epidemiologic category. We defined healthcare-associated infections as those cultured 48 hours or more after hospital admission [1, 19, 25]. We defined healthcare-associated community onset infections as those diagnosed prior to or within 48 hours of hospital admission with a history in the 12 months prior to infection of hemodialysis, hospitalization, surgery, or overnight stay in a care facility, or the presence of an indwelling intravascular catheter at the time of culture. We defined community-associated infections as those cultured prior to or within 48 hours of hospital admission in subjects without any of these healthcare exposures. We also determined insurance status information and International Classification of Diseases, 10th Edition (ICD-10) codes for calculation of the Elixhauser Comorbidity Index [26, 27] abstracted from Epic Clarity (Epic Systems, Veronia, Wisconsin).

### Outcomes

We determined outcomes via manual adjudication of patient records. The primary outcome was in-hospital mortality or hospice discharge. Secondary outcomes included ICU admission, 7-day readmission, recurrent bacteremia within a year after index infection, Pitt bacteremia score (PBS), and metastatic complications, including infective endocarditis, epidural abscess, soft tissue abscess, mycotic aneurysm, osteomyelitis, septic pulmonary emboli, and other septic emboli.

### Statistical and Sensitivity Analysis

We used descriptive statistics to report baseline characteristics and outcomes and analyzed these via Wilcoxon rank-sum or *t*-tests for continuous variables and χ^2^ or Fisher's exact tests for categorical variables, as appropriate. We examined the association between methicillin resistance status and outcomes using multivariable logistic regression models, reporting adjusted odds ratios (aORs). We defined metastatic complications as any infectious focus outside of the bloodstream, including infective endocarditis. In the primary analysis, we did not include PBS or length of infection in outcome models, as we considered illness severity to be a potential mediator on the causal pathway between methicillin resistance and downstream clinical outcomes. We also did not include choice of antibiotic for definitive therapy, given the general lack of overlap between the two groups, as well as whether any procedure performed, as we felt this did not necessarily imply source control which was beyond the scope of manual abstraction. We included covariates by first screening them with individual univariable regressions, incorporating those with *P* < .20 into the multivariable model; regardless of univariable regression results, we included age [19, 28–31], sex [29, 32, 33], race [34, 35], and Elixhauser comorbidity index [29–31, 36], and assessed for collinearity of final variables. As a sensitivity analysis to test whether PBS could be an indicator of acuity at time of presentation due to an unobserved factor (eg, delayed presentation to care), we repeated models that included PBS. As another sensitivity analysis to assess the robustness of findings, we separately used propensity score matching modeling, utilizing the nearest method, a ratio of 1, and a caliper of 0.1, with acceptable covariate balance.

We employed 2-sided hypothesis tests with α = 0.05 and conducted all analyses using R version 4.6.0 (R Foundation for Statistical Computing, Vienna, Austria) [37]. We followed the Strengthening the Reporting of Observational Studies in Epidemiology reporting guideline [38].

### Ethic Statement

The University of Pennsylvania Institutional Review Board approved the study (protocol number 21-1416 [850306]). The requirement for informed patient consent was waived because this was a retrospective observational study involving minimal risk to participants.

## RESULTS

### Baseline Characteristics

Among 329 subjects with first-episode SAB, 140 (42.6%) had MRSA and 189 (57.4%) had MSSA (Table 1). Overall, the cohort had a mean age of 55.1 years, and 129 (39.2%) were female. Patients with MRSA bacteremia were younger and had lower BMI, less private/commercial insurance, more skin and soft tissue infectious (SSTI) sources, less statin use, more active IDU, and higher rates of prior surgery in the past year, care facility stay in the past year, and any prior cultured *S. aureus* infection.

**Table 1.** Baseline Characteristics of Patients With *Staphylococcus aureus* Bacteremia

Characteristic	MSSA (N = 189)	MRSA (N = 140)	Overall (N = 329)	*P* Value
Female sex	78 (41.3)	51 (36.4)	129 (39.2)	.37
Age, years	56.6 (16.5)	53.0 (16.1)	55.1 (16.4)	.04
Race				
Black	82 (43.4)	48 (34.3)	130 (39.5)	.22
White	87 (46.0)	77 (55.0)	164 (49.8)	
Other or unknown	20 (10.6)	15 (10.6)	35 (10.6)	
Hispanic/Latino or unknown ethnicity	7 (3.7)	7 (5.0)	14 (4.3)	.56
BMI (kg/m^2^)				
<25	72 (38.1)	72 (51.4)	144 (43.8)	.01
25–30	46 (24.3)	36 (25.7)	82 (24.9)	
≥30	71 (37.6)	32 (22.9)	103 (31.3)	
Elixhauser comorbidity index	24.0 (12.2–34.0)	21.0 (11.0–32.0)	23.0 (11.0–33.0)	.20
Insurance				
Private/commercial	54 (28.6)	19 (13.6)	73 (22.2)	.003
Public, managed	91 (48.1)	89 (63.6)	180 (54.7)	
Public, unmanaged or uninsured/self-pay	44 (23.3)	32 (22.9)	76 (23.1)	
Admission to HUP versus PPMC	111 (58.7)	73 (52.1)	184 (55.9)	.23
Source				
SSTI	87 (46.0)	90 (64.3)	177 (53.8)	.005
Endovascular/access-related	45 (23.8)	27 (19.3)	72 (21.9)	
Deep-seated/visceral	14 (7.4)	8 (5.7)	22 (6.7)	
Other/unknown	43 (22.8)	15 (10.7)	58 (17.6)	
Presence of indwelling intravascular catheter or foreign body	54 (28.6)	41 (29.3)	95 (28.9)	. 89
Indwelling intravascular catheter	40 (21.2)	36 (25.7)	76 (23.1)	.33
Foreign body	18 (9.5)	13 (9.3)	31 (9.4)	.94
Length of episode, days	1.0 (1.0–3.0)	2.0 (1.0–5.0)	1.0 (1.0–4.0)	.07
Procedure performed	49 (25.9)	43 (30.7)	92 (28.0)	.34
Definitive antibiotic for treatment				
Cefadroxil	1 (0.5)	0 (0.0)	1 (0.3)	<.001
Cefazolin	134 (71.7)	0 (0.0)	134 (41.2)	
Cefazolin/Vancomycin	1 (0.5)	0 (0.0)	1 (0.3)	
Ceftaroline/Daptomycin	1 (0.5)	1 (0.7)	2 (0.6)	
Dalbavancin	0 (0.0)	3 (2.2)	3 (0.9)	
Daptomycin	6 (3.2)	27 (19.6)	33 (10.2)	
Nafcillin	5 (2.7)	0 (0.0)	5 (1.5)	
Vancomycin	39 (20.9)	107 (77.5)	146 (44.9)	
Statin use	89 (47.1)	48 (34.3)	137 (41.6)	.02
Anticoagulant use	68 (36.0)	50 (35.7)	118 (35.9)	.96
Hemodialysis in past 12 m	25 (13.2)	23 (16.4)	48 (14.6)	.42
Hospitalization in past 12 m	117 (61.9)	98 (70.0)	215 (65.3)	.13
Surgery in past 12 m	38 (20.1)	58 (41.4)	96 (29.2)	<.001
Overnight stay at a care facility in past 12 m	11 (5.8)	21 (15.0)	32 (9.7)	.005
Immunosuppression in past 6 m	28 (14.8)	13 (9.3)	41 (12.5)	.13
History of *S. aureus* infection^a^	18 (9.5)	39 (27.9)	57 (17.3)	<.001
Active injection drug use	22 (11.6)	48 (34.3)	70 (21.3)	<.001
Onset classification				
CA	45 (23.8)	31 (22.1)	76 (23.1)	.04
HACO	88 (46.6)	83 (59.3)	171 (52.0)	
HO	56 (29.6)	26 (18.6)	82 (24.9)	

Continuous variables are shown as mean (standard deviation) or median (interquartile range) based on distribution, and categorical variables are shown as count (%). Wilcoxon rank-sum tests were used for continuous variables as appropriate. χ^2^ or Fisher's exact tests were used for categorical variables as appropriate. Statistical significance was determined at two-sided α = .05. Procedures performed included debridement, incision and drainage, valve repair/replacement/debridement, foreign body removal, and other procedure not specified. Immunosuppression was defined as any use of immunosuppressants within 6 m prior to the index positive culture. Hemodialysis, hospitalization, surgery, and long-term care residence were indicated as true if within 12 m prior to the index *S. aureus* blood culture.

Abbreviations: BMI, body mass index; CA, community-acquired; HACO, healthcare-associated community onset; HO, hospital onset; HUP, Hospital of the University of Pennsylvania; MRSA, methicillin-resistant *Staphylococcus aureus*; MSSA, methicillin-susceptible *Staphylococcus aureus*; PPMC, Penn Presbyterian Medical Center; SSTI, skin and soft tissue infection.

^a^Indicates history of any *S. aureus* culture obtained at any anatomic site within the Penn Medicine/University of Pennsylvania Health System.

### Clinical Outcomes

Overall, in-hospital mortality or hospice discharge occurred in 19.1% of the cohort (Table 2). ICU admission, 7-day readmission, any metastatic complication, and recurrent bacteremia occurred in 40.7%, 6.1%, 26.1%, and 7.3% of the cohort, respectively. The mean PBS was 1.2. Those with MRSA had higher rates of soft tissue abscess and recurrent bacteremia and a lower mean PBS but were otherwise similar to those with MSSA.

**Table 2.** Outcomes of Patients With *Staphylococcus aureus* Bacteremia

Outcome	MSSA (N = 186)	MRSA (N = 143)	Overall (N = 329)	*P* Value
In-hospital mortality or hospice discharge	40 (21.2)	23 (16.4)	63 (19.1)	.28
Intensive care unit admission	82 (43.4)	52 (37.1)	134 (40.7)	.25
7-day readmission	9 (4.8)	11 (7.9)	20 (6.1)	.25
Any metastatic complication	44 (23.3)	42 (30.0)	86 (26.1)	.17
Infective endocarditis	19 (10.1)	14 (10.0)	33 (10.0)	.99
Epidural abscess	17 (9.0)	12 (8.6)	29 (8.8)	.89
Soft tissue abscess	3 (1.6)	12 (8.6)	15 (4.6)	.003
Mycotic aneurysm	0 (0.0)	1 (0.7)	1 (0.3)	.43
Osteomyelitis	8 (4.2)	5 (3.6)	13 (4.0)	.76
Septic pulmonary emboli	15 (7.9)	15 (10.7)	30 (9.1)	.39
Other septic emboli	0 (0.0)	2 (1.4)	2 (0.6)	.18
Recurrent bacteremia	9 (4.8)	15 (10.7)	24 (7.3)	.04
Pitt bacteremia score	1.5 (2.1)	0.9 (1.7)	1.2 (2.0)	.002

Continuous variables are shown as mean (standard deviation) and categorical variables are shown as count (%). The *t*-tests were used for continuous variables as appropriate. χ^2^ or Fisher's exact tests were used for categorical variables as appropriate. Statistical significance was determined at two-sided α = .05.

Abbreviations: MRSA, methicillin-resistant *Staphylococcus aureus*; MSSA, methicillin-susceptible *Staphylococcus aureus*.

### Multivariable Analysis Comparing MRSA and MSSA Bacteremia

After screening for potentially significant covariates in univariable regression and adjusting for these in multivariable regression, methicillin resistance was not associated with in-hospital mortality or hospice discharge (aOR 1.03, 95% CI 0.49–2.12, *P* = .94) (Table 3), nor was it associated with ICU admission (aOR 1.09, 95% CI 0.61–1.94, *P* = .78), 7-day readmission (aOR 1.51, 95% CI 0.51–4.67, *P* = .46), any metastatic complication (aOR 0.98, 95% CI 0.47–2.05, *P* = .94), or infective endocarditis (aOR 1.02, 95% CI 0.42–2.45, *P* = .96), but there was a trend toward significance for incidence of recurrent bacteremia (aOR 2.43, 95% CI 0.92–6.76, *P* = .08) (Supplementary Tables 1–5).

**Table 3.** Univariable and Multivariable Regression of In-hospital Morality or Hospice Discharge

Exposure or Covariate	Unadjusted OR	*P* Value	Adjusted OR	*P* Value
MRSA versus MSSA	0.73 (0.41–1.28)	.28	1.03 (0.49–2.12)	.94
Female sex	1.95 (1.12–3.40)	.02	1.99 (0.98–4.09)	.06
Age, years	1.04 (1.02–1.06)	<.001	1.02 (1.00–1.05)	.08
Race (reference: Black)				
White	1.07 (0.60–1.94)	.82	1.53 (0.70–3.42)	.29
Other/unknown	1.10 (0.41–2.72)	.84	1.03 (0.30–3.24)	.96
Hispanic/Latino or unknown ethnicity	0.31 (0.02–1.62)	.27	…	…
BMI (kg/m^2^) (reference: <25)				
25–30	1.10 (0.54–2.18)	.79	…	…
>30	1.16 (0.61–2.20)	.65	…	…
Elixhauser comorbidity index	1.08 (1.06–1.11)	<.001	1.08 (1.05–1.10)	<.001
Insurance (reference: Private/commercial)				
Public, managed	0.74 (0.38–1.48)	.39	…	…
Public, unmanaged or uninsured/self-pay	0.95 (0.43–2.09)	.90	…	…
Admission to HUP versus PPMC	1.47 (0.84–2.63)	.18	1.04 (0.50–2.17)	.92
Source (reference: SSTI)				
Endovascular/access-related	3.23 (1.62–6.48)	<.001	2.00 (0.82–4.88)	.12
Deep-seated/visceral	2.31 (0.70–6.59)	.14	0.96 (0.25–3.26)	.95
Other/unknown	3.25 (1.56–6.78)	.002	2.35 (0.94–5. 84)	.07
Presence of indwelling intravascular catheter	1.30 (0.68–2.39)	.42	…	…
Presence of foreign body	2.20 (0.94–4.85)	.06	0.87 (0.28–2.48)	.80
Statin use	0.90 (0.51–1.58)	.73	…	…
Anticoagulant or aspirin use	1.04 (0.58–1.82)	.91	…	…
Hemodialysis in past 12 m	1.13 (0.51–2.34)	.75	…	…
Hospitalization in past 12 m	0.99 (0.56–1.78)	.96	…	…
Surgery in past 12 m	0.87 (0.46–1.59)	.67	…	…
Overnight stay at a care facility in past 12 m	2.47 (1.09–5.35)	.02	1.86 (0.69–4.84)	.21
History of immunosuppression in past 6 m	2.88 (1.40–5.80)	.003	1.09 (0.42–2.71)	.86
History of *S. aureus* infection^a^	1.01 (0.47–2.03)	.97	…	…
Active injection drug use	0.33 (0.12–0.75)	.01	0.73 (0.20–2.49)	.63

Univariable regression was performed for all covariates listed individually; those with *P* value < 0.2 were included in the multivariable regression model, with the exceptions of methicillin resistance status, female sex, age, race, and Elixhauser comorbidity index, which were considered to be central to our research question and/or integral in the causal pathway and were thus included regardless of univariable regression results. Statistical significance was determined at two-sided α = .05. Immunosuppression was defined as any use of immunosuppressants within 6 m prior to the index *S. aureus* blood culture. Hemodialysis, hospitalization, surgery, and overnight stay at a care facility were indicated as true if within 12 m prior to the index *S. aureus* blood culture.

Abbreviations: BMI, body mass index; HUP, Hospital of the University of Pennsylvania; MRSA, methicillin-resistant *Staphylococcus aureus*; MSSA, methicillin-susceptible *Staphylococcus aureus*; OR, odds ratio; PPMC, Penn Presbyterian Medical Center; SSTI, skin and soft tissue infection.

^a^Indicates history of any *S. aureus* culture obtained at any anatomic site within the Penn Medicine/University of Pennsylvania Health System.

Among covariates, we found no collinearity in final models. Only Elixhauser comorbidity index was associated with increased in-hospital mortality or hospice discharge (aOR 1.08, 95% 1.05–1.10, *P* < .001) (Table 3). ICU admission was associated with Elixhauser comorbidity index (aOR 1.07, 95% 1.05–1.10, *P* < .001), unmanaged public insurance or lack of insurance/self-pay (aOR 0.27, 95% 0.11–0.65, *P* = .004), other/unknown source of infection (aOR 2.40, 95% 1.12–5.22, *P* = .02), and hospitalization in the past year (aOR 0.52, 95% 0.29–0.93, *P* = .03) (Supplementary Table 1). Seven-day readmission was associated with admission to HUP versus PPMC (aOR 0.34, 95% 0.10–0.97, *P* = .05) (Supplementary Table 2). Any metastatic complication was associated with managed public insurance (aOR 0.43, 95% 0.20–0.89, *P* = .02), any prior *S. aureus* infection (aOR 2.53, 95% 1.23–5.21, *P* = .01), and active IDU (aOR 4.49, 95% 1.80–11.67, *P* = .002) (Supplementary Table 3). Infective endocarditis was associated with unmanaged public insurance or lack of insurance/self-pay (aOR 0.19, 95% 0.04–0.71, *P* = .02), managed public insurance (aOR 0.37, 95% 0.14–0.92, *P* = .03), foreign body presence (aOR 3.47, 95% 1.03–10.87, *P* = .04), and active IDU (aOR 3.47, 95% 1.11–11.10, *P* = .03) (Supplementary Table 4). Recurrent bacteremia was associated with both white (aOR 0.31, 95% 0.11–0.79, *P* = .02) and other/unknown race (aOR 0.10, 95% 0.00–0.63, *P* = .04), managed public insurance (aOR 0.22, 95% 0.07–0.69, *P* = .009), and prior *S. aureus* infection (aOR 4.25, 95% 1.55–11.70, *P* = .005) (Supplementary Table 5). Of note, some univariable models failed to converge because of sparse events, and insurance status may reflect unmeasured social, behavioral, and healthcare access factors rather than a direct causal effect.

In the sensitivity analysis that included PBS in the model, methicillin resistance was not significantly associated with in-hospital mortality or hospice discharge (aOR 1.26, 95% CI 0.58–2.73, *P* = .56), ICU admission (aOR 1.19, 95% CI 0.66–2.15, *P* = .57), 7-day readmission (aOR 1.39, 95% CI 0.46–4.32, *P* = .56), any metastatic complication (aOR 0.98, 95% CI 0.52–1.82, *P* = .94), infective endocarditis (aOR 1.07, 95% CI 0.44–2.59, *P* = .88), or recurrent bacteremia (aOR 2.21, 95% CI 0.82–6.24, *P* = .12). PBS itself was associated with in-hospital mortality or hospice discharge (aOR 1.33, 95% CI 1.14–1.56, *P* < .001) and ICU admission (aOR 1.18, 95% CI 1.02–1.37, *P* = .03), but not 7-day readmission (aOR 0.72, 95% CI 0.40–1.10, *P* = .19), any metastatic complication (aOR 1.00, 95% CI 0.86–1.15, *P* = .97), infective endocarditis (aOR 1.09, 95% CI 0.89–1.32, *P* = .37), or recurrent bacteremia (aOR 0.78, 95% CI 0.54–1.02, *P* = .12).

### Propensity Score Matching

Propensity score matching resulted in 83 subjects each for both MRSA and MSSA bacteremia with standard mean difference of 1.30 and variance ratio of 1.07 (Supplementary Table 6 and Supplementary Figure 1). Methicillin resistance was not associated with in-hospital mortality or hospice discharge (aOR 0.84, 95% CI 0.37–1.90, *P* = .68), ICU admission (aOR 0.78, 95% CI 0.41–1.45, *P* = .43), 7-day readmission (aOR 1.66, 95% CI 0.53–5.72, *P* = .39), any metastatic complication (aOR 1.00, 95% CI 0.50–2.00, *P* = .99), infective endocarditis (aOR 0.86, 95% CI 0.29–2.52, *P* = .79), or recurrent bacteremia (aOR 2.84, 95% CI 0.79–13.35, *P* = .13).

## DISCUSSION

In this retrospective cohort study of all sequential adults with first-episode SAB at two academic hospitals, methicillin resistance was not associated with worse short-term clinical outcomes. Specifically, after adjustment for demographic factors, comorbidity burden, healthcare exposures, and infection characteristics, MRSA bacteremia was not associated with in-hospital mortality or hospice discharge, ICU admission, 7-day readmission, metastatic complications, infective endocarditis, or recurrent bacteremia within a year. These findings were consistent when tested by 2 separate analytic approaches, multivariable regression (both with and without PBS as a covariate) and propensity score-matched analyses. However, MRSA bacteremia was associated with distinct epidemiologic and clinical characteristics, including younger age, lower BMI, higher prevalence of IDU, greater likelihood that SSTI was the source of bacteremia, and lower severity of illness at presentation. Despite significant differences in baseline patient and infection characteristics in MRSA versus MSSA patients, these differences did not result in worse clinical outcomes. These observations suggest that MRSA bacteremia, at least in our setting, represents a distinct epidemiologic phenotype rather than a more severe disease state.

Historically, worse outcomes observed in MRSA compared with MSSA bacteremia have been attributed to several interrelated mechanisms. For example, earlier studies documented delays in effective antibiotic therapy for MRSA, reflecting use of empiric regimens that inadequately covered MRSA as well as limitations in rapid diagnostic testing [6, 7, 22, 23, 39]. Treatment options for MRSA have also historically been and now remain constrained, with vancomycin long serving as the standard of care despite well-described limitations, including slower bactericidal activity, inferior tissue penetration, and greater nephrotoxicity compared with β-lactam therapy for MSSA [40]. It has been noted that inadequate dosing of vancomycin commonly occurs as initial optimal dosage is difficult to predict for any individual [41–43]. Additionally, in previous studies MRSA has often been disproportionately concentrated among older patients with greater comorbidity burdens and more healthcare-associated exposures, confounding the relationship between methicillin resistance and poor outcomes [7, 22, 44, 45]. Differences in intrinsic virulence between MRSA and MSSA strains have been proposed [22], although available evidence remains inconsistent [46–49] and may reflect heterogeneity at the strain level rather than resistance phenotype alone [50].

Our findings suggest that many of the historical reasons cited for worse outcomes with MRSA may be less relevant in contemporary practice. In the present cohort, MRSA bacteremia was more common among younger patients with lower comorbidity burden, higher prevalence of IDU, and a predominance of SSTI as a source, in addition to lower severity of illness at presentation. These features are consistent with the increasing contribution of community-associated MRSA lineages (particularly the USA300 strain in the USA) to invasive disease and mirror epidemiologic trends reported in other studies during the past 20 years [10, 14, 51–54]. At the same time, recent advances in the standardized management of SAB may have attenuated differences in outcomes between MRSA and MSSA bacteremia [1, 19–21]. These shifts provide a biologically and clinically plausible explanation for the absence of outcome disparities observed in our study.

Notably, methicillin resistance was not independently associated with metastatic complications or infective endocarditis. Instead, these outcomes were associated with host and exposure-related factors, including IDU and the presence of implanted foreign bodies. This finding challenges the traditional perception that MRSA bacteremia intrinsically confers higher endovascular risk [55, 56] and contradicts other studies that have found that MSSA has higher risk of endocarditis than does MRSA [22, 57]. Our findings suggest that resistance phenotype alone may be a poor surrogate for predicting metastatic complications and infective endocarditis, with patient behaviors, structural risk factors, and infectious source appearing to play a more important role in contemporary SAB.

Although methicillin resistance was not independently associated with recurrent bacteremia in adjusted analyses, we did observe a nonsignificant trend toward increased recurrence with MRSA, both in the primary analysis and the propensity score-matched model. However, prior *S. aureus* infection cultured at our center was the strongest predictor of recurrence. This association may reflect persistent colonization with a virulent strain, ongoing exposure risk, or unmeasured behavior or environmental factors, particularly among those with IDU. Given the wide confidence intervals surrounding this estimate, these findings should be interpreted cautiously.

While determination of methicillin resistance status in *S. aureus* remains essential for guiding antibiotic selection and, when relevant, infection prevention interventions, our findings support a shift in prognostic assessment and risk stratification for consideration of preventive interventions focusing on host characteristics, comorbidity burden, illness severity, and infectious source.

This study has several limitations. First, its retrospective design introduces the potential for unmeasured, residual confounding. Second, we derived our findings from a single healthcare system, potentially limiting generalizability. Third, we lacked detailed microbiologic data, specifically genomic data such as strain typing and virulence factor profiling, which could further elucidate heterogeneity within our cohort. Fourth, while our cohort was relatively large and included sequential SAB patients, statistical power was limited for rare outcomes such as infective endocarditis and recurrent bacteremia. Fifth, the number of variables and event rates in the final models may have resulted in some degree of model instability, as evidenced by somewhat wide confidence intervals for certain variables (eg, active IDU for endocarditis); however, we used propensity score matching as a sensitivity analysis to address this and found conclusions regarding the primary analysis remained unchanged. Sixth, we did not include certain outcomes of interest, notably mortality outside the hospital after discharge (eg, 30-day mortality, 90-day mortality). Nonetheless, the consistency of findings across multiple outcomes and analytic approaches strengthens the validity of our conclusions to our primary research question.

## CONCLUSIONS

In this contemporary cohort of adults at a single academic medical center with first-episode SAB, methicillin resistance was associated with distinct epidemiologic and clinical characteristics but not with worse short-term outcomes, including mortality, ICU admission, readmission, metastatic complications, infective endocarditis, or recurrent infection. This contradicts outcome disparities historically attributed to methicillin resistance, though our data is more recent and therefore may reflect possible secular changes, such as differences in patient populations, illness severity, and clinical context, rather than resistance phenotype itself. As the epidemiology of SAB continues to evolve, risk stratification and management strategies should increasingly emphasize host factors, severity of illness, and infection source rather than methicillin resistance alone.

## Supplementary Material

ofag354_Supplementary_Data

## Supplementary Data

Supplementary materials are available at *Open Forum Infectious Diseases* online. Consisting of data provided by the authors to benefit the reader, the posted materials are not copyedited and are the sole responsibility of the authors, so questions or comments should be addressed to the corresponding author.
