# Supplementary material for: Impact of Methicillin Resistance on Outcomes of Staphylococcus aureus Bacteremia at a U.S. Center, 2023–2024
Source: Open Forum Infect Dis. 2026 Jun 6;13(6):ofag354. doi: 10.1093/ofid/ofag354 (PMC13263529; doi:10.1093/ofid/ofag354)
Supplement: ofag354_Supplementary_Data [file ofag354_supplementary_data.docx]

# **Supplement**

**Impact of Methicillin Resistance on Outcomes of *Staphylococcus aureus* Bacteremia at a U.S. Center, 2023–2024**

Joseph B. Ladines-Lim, MD, PhD^1,2,3^, Aidan Nemiroff,^4^ Leigh Cressman, MA^3^, Michael Z. David, MD, PhD^1,3,5^

**Affiliations:**

^1^ Division of Infectious Diseases, Department of Medicine, Penn Medicine, Perelman School of Medicine, University of Pennsylvania, Philadelphia, Pennsylvania, United States of America

^2^ Leonard Davis Institute of Health Economics, University of Pennsylvania, Philadelphia, Pennsylvania, United States of America

^3^ Center for Clinical Epidemiology and Biostatistics, Perelman School of Medicine, University of Pennsylvania, Philadelphia, Pennsylvania, United States of America

^4^ The College of Arts & Sciences, University of Pennsylvania, Philadelphia, Pennsylvania, United States of America

^5^ Department of Biostatistics and Epidemiology, Perelman School of Medicine, University of Pennsylvania, Philadelphia, Pennsylvania, United States of America

**Address correspondence to:** Joseph B. Ladines-Lim, MD, PhD, 3400 Spruce St, 3 Silverstein, Ste E, Philadelphia, Pennsylvania, United States of America, 19104-4238. Email: [joseph.ladines-lim@pennmedicine.upenn.edu](mailto:joseph.ladines-lim@pennmedicine.upenn.edu). Phone: 267-581-2092.

**Date:** May 15, 2026

# **Table of Contents**

**Supplementary Figure 1. Love plot of covariates in propensity score matching between methicillin-resistant and methicillin-susceptible *Staphylococcus aureus* bacteremia cohorts.**

**Supplementary Table 1. Univariable and multivariable regression with intensive care admission.**

**Supplementary Table 2. Univariable and multivariable regression of 7-day readmission.**

**Supplementary Table 3. Univariable and multivariable regression of any metastatic complication.**

**Supplementary Table 4. Univariable and multivariable regression of infective endocarditis.**

**Supplementary Table 5. Univariable and multivariable regression of recurrent bacteremia.**

**Supplementary Table 6. Propensity score matching balance diagnostics for methicillin-resistant and methicillin-susceptible *Staphylococcus aureus* bacteremia cohorts.**

# **Supplementary Figure 1. Love plot of covariates in propensity score matching between methicillin-resistant and methicillin-susceptible *Staphylococcus aureus* bacteremia cohorts.**


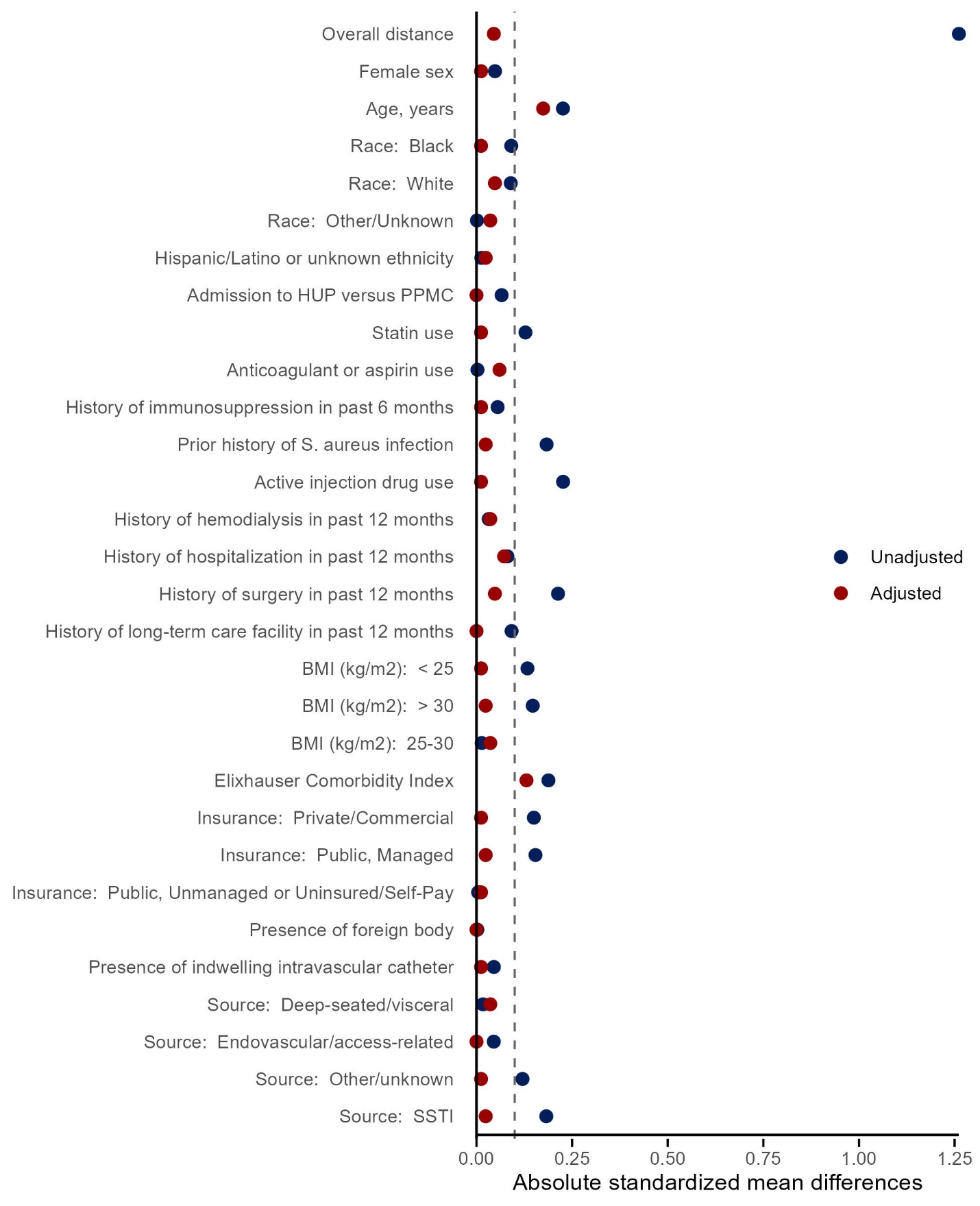


Propensity score matching was performed using the nearest method, ratio of 1, and caliper of 0.1. Body mass index (BMI) is shown in units of kg/m^2^. Prior history of *S. aureus* infection was defined as any *S. aureus* culture obtained at any anatomic site within the Penn Medicine/University of Pennsylvania Health System.

Abbreviations: BMI, body mass index; HUP, Hospital of the University of Pennsylvania; PPMC, Penn Presbyterian Medical Center; SSTI, skin and soft tissue infection.

# **Supplementary Table 1.** **Univariable and multivariable regression of intensive care admission.**

| **Exposure or Covariate** | **Unadjusted OR** | **P value** | **Adjusted OR** | **P value** |
| --- | --- | --- | --- | --- |
| **MRSA versus MSSA** | 0.77 (0.49–1.20) | .25 | 1.09 (0.61–1.94) | .78 |
| **Female sex** | 1.02 (0.65–1.61) | .92 | 0.79 (0.44–1.39) | .41 |
| **Age, years** | 1.02 (1.00–1.03) | .03 | 1.00 (0.98–1.02) | .96 |
| **Race (reference: Black)** |  | | | |
| White | 0.95 (0.60–1.53) | .84 | 1.52 (0.82–2.89) | .19 |
| Other/Unknown | 1.22 (0.57–2.59) | .60 | 1.34 (0.53–3.38) | .53 |
| **Hispanic/Latino or unknown ethnicity** | 1.48 (0.50–4.42) | .47 | … | … |
| **BMI (kg/m^2^) (reference: < 25)** |  | | | |
| 25–30 | 0.98 (0.56–1.72) | .95 | 1.03 (0.51–2.04) | .94 |
| > 30 | 1.41 (0.85–2.36) | .19 | 1.84 (0.95–3.61) | .07 |
| **Elixhauser Comorbidity Index** | 1.06 (1.05–1.08) | <.001 | 1.07 (1.05–1.10) | <.001 |
| **Insurance (reference: Private/commercial)** |  | | | |
| Public, managed | 0.68 (0.39–1.17) | .17 | 0.90 (0.44–1.87) | .78 |
| Public, unmanaged or uninsured/self-pay | 0.42 (0.21–0.82) | .01 | 0.27 (0.11–0.65) | .004 |
| **Admission to HUP versus PPMC** | 1.00 (0.64–1.56) | .99 | … | … |
| **Source (reference: SSTI)** |  | | | |
| Endovascular/access-related | 1.47 (0.84–2.58) | .17 | 0.69 (0.32–1.44) | .33 |
| Deep-seated/visceral | 1.35 (0.53–3.31) | .52 | 0.63 (0.20–1.89) | .41 |
| Other/unknown | 2.76 (1.51–5.12) | .001 | 2.40 (1.12–5.22) | .02 |
| **Presence of indwelling intravascular catheter** | 1.00 (0.59–1.69) | .99 | … | … |
| **Presence of foreign body** | 2.93 (1.38–6.56) | .006 | 2.53 (0.98–6.82) | .06 |
| **Statin use** | 1.18 (0.76–1.84) | .47 | … | … |
| **Anticoagulant or aspirin use** | 1.72 (1.09–2.72) | .02 | 1.54 (0.85–2.82) | .15 |
| **Hemodialysis in past 12 months** | 1.41 (0.76–2.61) | .27 | … | … |
| **Hospitalization in past 12 months** | 0.74 (0.46–1.17) | .19 | 0.52 (0.29–0.93) | .03 |
| **Surgery in past 12 months** | 1.19 (0.73–1.93) | .47 | … | … |
| **Overnight stay at a care facility in past 12 months** | 2.01 (0.96–4.26) | .06 | 1.57 (0.63–4.00) | .34 |
| **History of immunosuppression in past 6 months** | 0.92 (0.46–1.79) | .81 | … | … |
| **History of *S. aureus* infection*** | 0.68 (0.37–1.23) | .21 | … | … |
| **Active injection drug use** | 0.55 (0.31–0.96) | .04 | 0.80 (0.32–2.00) | .63 |

* Indicates history of any *S. aureus* culture obtained at any anatomic site within the Penn Medicine/University of Pennsylvania Health System.
Univariable regression was performed for all covariates listed individually; those with p value less than 0.2 were included in the multivariable regression model, with the exceptions of methicillin resistance status, female sex, age, race, and Elixhauser Comorbidity Index, which were considered to be central to our research question and/or integral in the causal pathway and were thus included regardless of univariable regression results. Statistical significance was determined at two-sided ɑ = 0.05. Immunosuppression was defined as any use of immunosuppressants within 6 months prior to the index *S. aureus* blood culture. Hemodialysis, hospitalization, surgery, and overnight stay at a care facility were indicated as true if within 12 months prior to the index *S. aureus* blood culture.
Abbreviations: BMI, body mass index; HUP, Hospital of the University of Pennsylvania; MRSA, methicillin-resistant *S. aureus*; MSSA, methicillin-susceptible *S. aureus*; OR, odds ratio; PPMC, Penn Presbyterian Medical Center; SSTI, skin and soft tissue infection.

# **Supplementary Table 2. Univariable and multivariable regression of 7-day readmission.**

| **Exposure or Covariate** | **Unadjusted OR** | **P value** | **Adjusted OR** | **P value** |
| --- | --- | --- | --- | --- |
| **MRSA versus MSSA** | 1.71 (0.69–4.34) | .25 | 1.51 (0.51–4.67) | .46 |
| **Female sex** | 1.98 (0.80–5.04) | .14 | 2.02 (0.73–5.76) | .18 |
| **Age, years** | 0.97 (0.94–1.00) | .04 | 0.99 (0.94–1.02) | .46 |
| **Race (reference: Black)** |  | | | |
| White | 1.10 (0.43–2.91) | .85 | 0.89 (0.26–3.05) | .85 |
| Other/Unknown | 0.45 (0.02–2.57) | .46 | 0.46 (0.02–3.36) | .51 |
| **Hispanic/Latino or unknown ethnicity** | … | … | … | … |
| **BMI (kg/m^2^) (reference: < 25)** |  | | | |
| 25–30 | 0.95 (0.32–2.61) | .93 | 1.20 (0.35–3.86) | .76 |
| > 30 | 0.36 (0.08–1.20) | .13 | 0.38 (0.07–1.57) | .21 |
| **Elixhauser Comorbidity Index** | 0.98 (0.95–1.01) | .17 | 0.99 (0.95–1.02) | .48 |
| **Insurance (reference: Private/commercial)** |  | | | |
| Public, managed | … | … | 1.63 (0.41–8.56) | .52 |
| Public, unmanaged or uninsured/self-pay | … | … | … | … |
| **Admission to HUP versus PPMC** | 0.32 (0.11–0.81) | .02 | 0.34 (0.10–0.97) | .05 |
| **Source (reference: SSTI)** |  | | | |
| Endovascular/access-related | 0.74 (0.20–2.18) | .61 | 5.36 (0.89–31.09) | .06 |
| Deep-seated/visceral | 1.26 (0.19–5.02) | .77 | 4.79 (0.54–34.45) | .12 |
| Other/unknown | 0.22 (0.01–1.15) | .15 | 0.44 (0.02–2.96) | .47 |
| **Presence of indwelling intravascular catheter** | 0.35 (0.06–1.26) | .17 | 0.20 (0.02–1.28) | .11 |
| **Presence of foreign body** | 0.49 (0.03–2.49) | .49 | … | … |
| **Statin use** | 1.16 (0.45–2.88) | .75 | … | … |
| **Anticoagulant or aspirin use** | 0.96 (0.35–2.42) | .93 | … | … |
| **Hemodialysis in past 12 months** | 0.29 (0.02–1.47) | .24 | … | … |
| **Hospitalization in past 12 months** | 0.98 (0.39–2.69) | .97 | … | … |
| **Surgery in past 12 months** | 1.67 (0.64–4.19) | .28 | … | … |
| **Overnight stay at a care facility in past 12 months** | 1.03 (0.16–3.82) | .97 | … | … |
| **History of immunosuppression in past 6 months** | 0.77 (0.12–2.81) | .73 | … | … |
| **History of *S. aureus* infection*** | 0.83 (0.19–2.59) | .78 | … | … |
| **Active injection drug use** | 3.33 (1.29–8.39) | .01 | 1.23 (0.30–5.07) | .78 |

* Indicates history of any *S. aureus* culture obtained at any anatomic site within the Penn Medicine/University of Pennsylvania Health System.

Univariable regression was performed for all covariates listed individually; those with p value less than 0.2 were included in the multivariable regression model, with the exceptions of methicillin resistance status, female sex, age, race, and Elixhauser Comorbidity Index, which were considered to be central to our research question and/or integral in the causal pathway and were thus included regardless of univariable regression results. Univariable regression failed to converge for insurance and Hispanic/Latino or unknown ethnicity. Statistical significance was determined at two-sided ɑ = 0.05. Immunosuppression was defined as any use of immunosuppressants within 6 months prior to the index *S. aureus* blood culture. Hemodialysis, hospitalization, surgery, and overnight stay at a care facility were indicated as true if within 12 months prior to the index *S. aureus* blood culture.
Abbreviations: BMI, body mass index; HUP, Hospital of the University of Pennsylvania; MRSA, methicillin-resistant *S. aureus*; MSSA, methicillin-susceptible *S. aureus*; OR, odds ratio; PPMC, Penn Presbyterian Medical Center; SSTI, skin and soft tissue infection.

# **Supplementary Table 3. Univariable and multivariable regression of any metastatic complication.**

| **Exposure or Covariate** | **Unadjusted OR** | **P value** | **Adjusted OR** | **P value** |
| --- | --- | --- | --- | --- |
| **MRSA versus MSSA** | 1.41 (0.86–2.32) | .17 | 0.98 (0.47–2.05) | .94 |
| **Female sex** | 1.09 (0.66–1.79) | .74 | 0.83 (0.45–1.53) | .56 |
| **Age, years** | 0.98 (0.97–1.00) | .03 | 1.02 (0.99–1.04) | .21 |
| **Race (reference: Black)** |  | | | |
| White | 1.49 (0.87–2.58) | .15 | 0.76 (0.38–1.51) | .44 |
| Other/Unknown | 2.25 (0.99–5.02) | .05 | 1.56 (0.60–3.94) | .35 |
| **Hispanic/Latino or unknown ethnicity** | 0.46 (0.07–1.73) | .31 | … | … |
| **BMI (kg/m^2^) (reference: < 25)** |  | | | |
| 25–30 | 0.95 (0.51–1.72) | .86 | 1.03 (0.51–2.09) | .93 |
| > 30 | 0.62 (0.34–1.12) | .12 | 1.08 (0.52–2.21) | .84 |
| **Elixhauser Comorbidity Index** | 1.00 (0.99–1.02) | .66 | 1.02 (1.00–1.03) | .11 |
| **Insurance (reference: Private/commercial)** |  | | | |
| Public, managed | 0.58 (0.33–1.06) | .07 | 0.43 (0.20–0.89) | .02 |
| Public, unmanaged or uninsured/self-pay | 0.48 (0.23–0.99) | .05 | 0.49 (0.20–1.20) | .12 |
| **Admission to HUP versus PPMC** | 0.68 (0.41–1.11) | .12 | 0.69 (0.39–1.24) | .22 |
| **Source (reference: SSTI)** |  | | | |
| Endovascular/access-related | 0.63 (0.32–1.19) | .17 | 1.42 (0.57–3.55) | .45 |
| Deep-seated/visceral | 0.53 (0.15–1.51) | .28 | 0.82 (0.20–2.73) | .76 |
| Other/unknown | 0.84 (0.42–1.61) | .61 | 1.09 (0.48–2.45) | .83 |
| **Presence of indwelling intravascular catheter** | 0.45 (0.22–0.86) | .02 | 0.80 (0.30–2.01) | .64 |
| **Presence of foreign body** | 1.39 (0.60–3.02) | .42 | … | … |
| **Statin use** | 0.48 (0.28–0.81) | .006 | 0.57 (0.27–1.16) | .12 |
| **Anticoagulant or aspirin use** | 0.66 (0.38–1.12) | .13 | 0.99 (0.51–1.93) | .99 |
| **Hemodialysis in past 12 months** | 0.36 (0.13–0.82) | .02 | 0.51 (0.17–1.33) | .19 |
| **Hospitalization in past 12 months** | 0.75 (0.45–1.25) | .27 | … | … |
| **Surgery in past 12 months** | 1.07 (0.62–1.82) | .80 | … | … |
| **Overnight stay at a care facility in past 12 months** | 0.63 (0.23–1.48) | .32 | … | … |
| **History of immunosuppression in past 6 months** | 0.27 (0.08–0.70) | .02 | 0.34 (0.09–1.06) | .09 |
| **History of *S. aureus* infection*** | 2.96 (1.63–5.38) | <.001 | 2.53 (1.23–5.21) | .01 |
| **Active injection drug use** | 4.43 (2.53–7.80) | <.001 | 4.49 (1.80–11.67) | .002 |

* Indicates history of any *S. aureus* culture obtained at any anatomic site within the Penn Medicine/University of Pennsylvania Health System.

Univariable regression was performed for all covariates listed individually; those with p value less than 0.2 were included in the multivariable regression model, with the exceptions of methicillin resistance status, female sex, age, race, and Elixhauser Comorbidity Index, which were considered to be central to our research question and/or integral in the causal pathway and were thus included regardless of univariable regression results. Statistical significance was determined at two-sided ɑ = 0.05. Immunosuppression was defined as any use of immunosuppressants within 6 months prior to the index *S. aureus* blood culture. Hemodialysis, hospitalization, surgery, and overnight stay at a care facility were indicated as true if within 12 months prior to the index *S. aureus* blood culture.
Abbreviations: BMI, body mass index; HUP, Hospital of the University of Pennsylvania; MRSA, methicillin-resistant *S. aureus*; MSSA, methicillin-susceptible *S. aureus*; OR, odds ratio; PPMC, Penn Presbyterian Medical Center; SSTI, skin and soft tissue infection.

# **Supplementary Table 4. Univariable and multivariable regression of infective endocarditis.**

| **Exposure or Covariate** | **Unadjusted OR** | **P value** | **Adjusted OR** | **P value** |
| --- | --- | --- | --- | --- |
| **MRSA versus MSSA** | 0.99 (0.47–2.05) | .99 | 1.02 (0.42–2.45) | .96 |
| **Female sex** | 1.52 (0.73–3.15) | .25 | 1.31 (0.58–2.96) | .51 |
| **Age, years** | 0.99 (0.97–1.01) | .36 | 1.01 (0.98–1.04) | .56 |
| **Race (reference: Black)** |  | | | |
| White | 1.21 (0.53–2.87) | .66 | 0.72 (0.27–1.91) | .50 |
| Other/Unknown | 3.56 (1.25–9.88) | .01 | 2.07 (0.62–6.57) | .22 |
| **Hispanic/Latino or unknown ethnicity** | 0.68 (0.04–3.59) | .71 | … | … |
| **BMI (kg/m^2^) (reference: < 25)** |  | | | |
| 25–30 | 1.56 (0.65–3.67) | .31 | … | … |
| > 30 | 0.96 (0.38–2.33) | .94 | … | … |
| **Elixhauser Comorbidity Index** | 1.02 (1.00–1.04) | .06 | 1.02 (0.99–1.05) | .12 |
| **Insurance (reference: Private/commercial)** |  | | | |
| Public, managed | 0.38 (0.17–0.85) | .02 | 0.37 (0.14–0.92) | .03 |
| Public, unmanaged or uninsured/self-pay | 0.23 (0.06–0.69) | .01 | 0.19 (0.04–0.71) | .02 |
| **Admission to HUP versus PPMC** | 0.72 (0.35–1.48) | .37 | … | … |
| **Source (reference: SSTI)** |  | | | |
| Endovascular/access-related | 1.54 (0.62–3.65) | .33 | … | … |
| Deep-seated/visceral | 0.51 (0.03–2.75) | .53 | … | … |
| Other/unknown | 1.73 (0.66–4.22) | .24 | … | … |
| **Presence of indwelling intravascular catheter** | 0.89 (0.34–2.03) | .79 | … | … |
| **Presence of foreign body** | 3.05 (1.13–7.47) | .02 | 3.47 (1.03–10.87) | .04 |
| **Statin use** | 0.78 (0.36–1.62) | .52 | … | … |
| **Anticoagulant or aspirin use** | 1.18 (0.55–2.45) | .66 | … | … |
| **Hemodialysis in past 12 months** | 0.56 (0.13–1.65) | .35 | … | … |
| **Hospitalization in past 12 months** | 0.60 (0.29–1.26) | .17 | 0.48 (0.20–1.11) | .09 |
| **Surgery in past 12 months** | 1.24 (0.56–2.62) | .58 | … | … |
| **Overnight stay at a care facility in past 12 months** | 0.92 (0.21–2.80) | .90 | … | … |
| **History of immunosuppression in past 6 months** | … | … | … | … |
| **History of *S. aureus* infection*** | 1.94 (0.81–4.30) | .12 | 1.71 (0.58–4.83) | .32 |
| **Active injection drug use** | 2.34 (1.06–4.98) | .03 | 3.47 (1.11–11.10) | .03 |

* Indicates history of any *S. aureus* culture obtained at any anatomic site within the Penn Medicine/University of Pennsylvania Health System.

Univariable regression was performed for all covariates listed individually; those with p value less than 0.2 were included in the multivariable regression model, with the exceptions of methicillin resistance status, female sex, age, race, and Elixhauser Comorbidity Index, which were considered to be central to our research question and/or integral in the causal pathway and were thus included regardless of univariable regression results. Univariable regression failed to converge for history of immunosuppression. Statistical significance was determined at two-sided ɑ = 0.05. Immunosuppression was defined as any use of immunosuppressants within 6 months prior to the index *S. aureus* blood culture. Hemodialysis, hospitalization, surgery, and overnight stay at a care facility were indicated as true if within 12 months prior to the index *S. aureus* blood culture.
Abbreviations: BMI, body mass index; HUP, Hospital of the University of Pennsylvania; MRSA, methicillin-resistant *S. aureus*; MSSA, methicillin-susceptible *S. aureus*; OR, odds ratio; PPMC, Penn Presbyterian Medical Center; SSTI, skin and soft tissue infection.

# **Supplementary Table 5. Univariable and multivariable regression of recurrent bacteremia.**

| **Exposure or Covariate** | **Unadjusted OR** | **P value** | **Adjusted OR** | **P value** |
| --- | --- | --- | --- | --- |
| **MRSA versus MSSA** | 2.40 (1.03–5.88) | .05 | 2.43 (0.92–6.76) | .08 |
| **Female sex** | 0.76 (0.30–1.78) | .54 | 0.75 (0.28–1.90) | .56 |
| **Age, years** | 0.99 (0.96–1.01) | .37 | 0.98 (0.95–1.01) | .29 |
| **Race (reference: Black)** |  | | | |
| White | 0.58 (0.24–1.37) | .22 | 0.31 (0.11–0.79) | .02 |
| Other/Unknown | 0.26 (0.01–1.40) | .21 | 0.10 (0.00–0.63) | .04 |
| **Hispanic/Latino or unknown ethnicity** | … | … | … | … |
| **BMI (kg/m^2^) (reference: < 25)** |  | | | |
| 25–30 | 1.25 (0.44–3.39) | .66 | … | … |
| > 30 | 0.98 (0.34–2.63) | .96 | … | … |
| **Elixhauser Comorbidity Index** | 1.01 (0.99–1.04) | .38 | 1.02 (0.99–1.05) | .18 |
| **Insurance (reference: Private/commercial)** |  | | | |
| Public, managed | 0.43 (0.16–1.18) | .09 | 0.22 (0.07–0.69) | .009 |
| Public, unmanaged or uninsured/self-pay | 0.82 (0.27–2.42) | .72 | 0.73 (0.20–2.55) | .62 |
| **Admission to HUP versus PPMC** | 0.93 (0.40–2.17) | .86 | … | … |
| **Source (reference: SSTI)** |  | | | |
| Endovascular/access-related | 0.68 (0.19–1.99) | .52 | … | … |
| Deep-seated/visceral | 0.55 (0.03–2.98) | .58 | … | … |
| Other/unknown | 1.10 (0.34–3.02) | .86 | … | … |
| **Presence of indwelling intravascular catheter** | 0.45 (0.11–1.36) | .21 | … | … |
| **Presence of foreign body** | 0.40 (0.02–2.00) | .38 | … | … |
| **Statin use** | 1.20 (0.51–2.77) | .67 | … | … |
| **Anticoagulant or aspirin use** | 1.30 (0.54–3.01) | .54 | … | … |
| **Hemodialysis in past 12 months** | 1.19 (0.33–3.32) | .76 | … | … |
| **Hospitalization in past 12 months** | 1.64 (0.67–4.65) | .31 | … | … |
| **Surgery in past 12 months** | 1.23 (0.48–2.91) | .64 | … | … |
| **Overnight stay at a care facility in past 12 months** | 1.36 (0.31–4.26) | .64 | … | … |
| **History of immunosuppression in past 6 months** | 0.62 (0.10–2.22) | .53 | … | … |
| **History of *S. aureus* infection*** | 3.92 (1.60–9.30) | .002 | 4.25 (1.55–11.70) | .005 |
| **Active injection drug use** | 1.58 (0.59–3.84) | .33 | … | … |

* Indicates history of any *S. aureus* culture obtained at any anatomic site within the Penn Medicine/University of Pennsylvania Health System.

Univariable regression was performed for all covariates listed individually; those with p value less than 0.2 were included in the multivariable regression model, with the exceptions of methicillin resistance status, female sex, age, race, and Elixhauser Comorbidity Index, which were considered to be central to our research question and/or integral in the causal pathway and were thus included regardless of univariable regression results. Univariable regression failed to converge for Hispanic/Latino or unknown ethnicity. Statistical significance was determined at two-sided ɑ = 0.05. Immunosuppression was defined as any use of immunosuppressants within 6 months prior to the index *S. aureus* blood culture. Hemodialysis, hospitalization, surgery, and overnight stay at a care facility were indicated as true if within 12 months prior to the index *S. aureus* blood culture.
Abbreviations: BMI, body mass index; HUP, Hospital of the University of Pennsylvania; MRSA, methicillin-resistant *S. aureus*; MSSA, methicillin-susceptible *S. aureus*; OR, odds ratio; PPMC, Penn Presbyterian Medical Center; SSTI, skin and soft tissue infection.

# **Supplementary Table 6. Propensity score matching balance diagnostics for methicillin-resistant and methicillin-susceptible *Staphylococcus aureus* bacteremia cohorts.**

| **Covariate** | **MRSA (before)** | **MSSA (before)** | **SMD** | **MRSA (after)** | **MSSA (after)** | **Variance ratio (after)** |
| --- | --- | --- | --- | --- | --- | --- |
| **Overall distance** | 0.59 | 0.30 | 1.30 | 0.47 | 0.46 | 1.07 |
| **Female sex** | 0.36 | 0.41 | -0.05 | 0.36 | 0.37 | … |
| **Age, years** | 52.95 | 56.63 | -0.23 | 53.72 | 56.57 | 0.94 |
| **Race: Black** | 0.34 | 0.43 | -0.09 | 0.33 | 0.34 | … |
| **Race: White** | 0.55 | 0.46 | 0.09 | 0.58 | 0.53 | … |
| **Race: Other/Unknown** | 0.11 | 0.11 | 0.00 | 0.10 | 0.13 | … |
| **Hispanic/Latino or unknown ethnicity** | 0.05 | 0.04 | 0.01 | 0.04 | 0.06 | … |
| **BMI: < 25 kg/m^2^** | 0.50 | 0.39 | 0.12 | 0.47 | 0.48 | … |
| **BMI: 25–30 kg/m^2^** | 0.24 | 0.37 | -0.13 | 0.26 | 0.29 | … |
| **BMI: > 30 kg/m^2^** | 0.26 | 0.24 | 0.02 | 0.27 | 0.22 | … |
| **Elixhauser Comorbidity Index** | 22.45 | 25.32 | -0.17 | 22.52 | 20.85 | 1.04 |
| **Insurance: Private/commercial** | 0.14 | 0.28 | -0.15 | 0.21 | 0.20 | … |
| **Insurance: Public, managed** | 0.64 | 0.48 | 0.16 | 0.56 | 0.56 | … |
| **Insurance: Public, unmanaged or uninsured/self-pay** | 0.22 | 0.24 | -0.01 | 0.22 | 0.24 | … |
| **Admission to HUP versus PPMC** | 0.52 | 0.59 | -0.07 | 0.55 | 0.55 | … |
| **Source: SSTI** | 0.64 | 0.46 | 0.18 | 0.57 | 0.54 | … |
| **Source: Endovascular/access-related** | 0.19 | 0.24 | -0.05 | 0.22 | 0.22 | … |
| **Source: Deep-seated/visceral** | 0.06 | 0.07 | -0.02 | 0.06 | 0.10 | … |
| **Source: Other/unknown** | 0.11 | 0.23 | -0.12 | 0.16 | 0.14 | … |
| **Presence of indwelling intravascular catheter** | 0.26 | 0.21 | 0.05 | 0.24 | 0.23 | … |
| **Presence of foreign body** | 0.09 | 0.10 | 0.00 | 0.10 | 0.10 | … |
| **Statin use** | 0.34 | 0.47 | -0.13 | 0.42 | 0.41 | … |
| **Anticoagulant or aspirin use** | 0.36 | 0.36 | 0.00 | 0.33 | 0.39 | … |
| **Hemodialysis in past 12 months** | 0.16 | 0.13 | 0.03 | 0.16 | 0.19 | … |
| **Hospitalization in past 12 months** | 0.70 | 0.62 | 0.08 | 0.66 | 0.59 | … |
| **Surgery in past 12 months** | 0.41 | 0.20 | 0.21 | 0.35 | 0.30 | … |
| **Overnight stay at a care facility in past 12 months** | 0.15 | 0.06 | 0.09 | 0.11 | 0.11 | … |
| **History of immunosuppression in past 6 months** | 0.09 | 0.15 | -0.06 | 0.16 | 0.14 | … |
| **History of *S. aureus* infection*** | 0.28 | 0.10 | 0.18 | 0.18 | 0.16 | … |
| **Active injection drug use** | 0.34 | 0.12 | 0.23 | 0.24 | 0.23 | … |

* Indicates history of any *S. aureus* culture obtained at any anatomic site within the Penn Medicine/University of Pennsylvania Health System.

Abbreviations: BMI, body mass index; HUP, Hospital of the University of Pennsylvania; MRSA, methicillin-resistant *S. aureus*; MSSA, methicillin-susceptible *S. aureus*; PPMC, Penn Presbyterian Medical Center; SMD, standard mean difference; SSTI, skin and soft tissue infection.
